# Semantic convergence in culturally loaded text translation by Large Language Models: a cross-model empirical analysis of English translations of *The Four Books*

**DOI:** 10.3389/fpsyg.2026.1829488

**Published:** 2026-05-14

**Authors:** Qizheng Wan, Qiangfu Yu

**Affiliations:** Faculty of Humanities and Foreign Languages, Xi’an University of Technology, Xi’an, Shaanxi, China

**Keywords:** cultural translation, Large Language Models, semantic consistency, semantic convergence, *The Four Books*

## Abstract

This study takes *The Four Books* as its corpus to construct a sentence-aligned parallel corpus. Employing sentence embeddings and cosine similarity, it systematically analyzes the semantic convergence of English translations of culturally loaded texts generated by four Large Language Models (LLMs): ChatGPT-5, Google Translate, Deepseek-V3.2, and Ernie Bot-5. The findings reveal that: (1) the translations from different models exhibit a high degree of overall semantic consistency, with the average cosine similarity for twenty core Confucian concepts all exceeding 0.73, indicating a significant trend of cross-model semantic convergence; (2) there are notable differences in stability among concepts, with those having clear referents and well-defined semantic boundaries demonstrating higher stability, while abstract concepts with greater interpretive latitude show more pronounced divergence; (3) systematic strategic divergences exist among the LLMs, with pairwise similarity distributions revealing differing orientations between cultural preservation and functional interpretation. Furthermore, analysis of cosine similarity identified that low-similarity outliers primarily stem from semantic divergence of polysemous words, differences in handling cultural-specific items, divergent translation strategies, and local context misinterpretations, reflecting the mechanisms of semantic variation in specific contexts. Grounded in the internal structure of the text, this study proposes a multi-layered analytical framework for cross-model semantic convergence, “sentence-level alignment– vector computation– concept aggregation,” providing methodological support for quantitative research on LLM translation of culturally loaded texts. It offers an empirical foundation for understanding the capability boundaries, strategic orientations, and potential risks of cultural meaning simplification in LLMs’ cross-cultural semantic representations.

## 1 Introduction

The rapid development of LLMs is profoundly reshaping the landscape of machine translation. While traditional Neural Machine Translation (NMT) demonstrates significant advantages in context modeling and semantic representation (Stahlberg, 2020), its performance remains constrained by factors such as corpus size, cross-linguistic structural differences, and domain shift. In contrast, LLMs exhibit notable strengths in contextual understanding and output fluency (Jiao et al., 2023; Son and Kim, 2023; AlAfnan, 2024). However, the question remains whether this superiority holds when processing texts rich in culture-specific concepts. Existing studies indicate that the translation quality of LLMs can be inconsistent when dealing with literary or culturally dense texts (Gao et al., 2024; Farghal and Haider, 2024), raising a critical question: When faced with highly abstract and “untranslatable” core concepts from *The Four Books* (*The Great Learning*, *The Doctrine of the Mean*, *The Analects*, and *Mencius*), the core canon of Confucian thought, such as “仁” (rén, benevolence), “义” (yì, righteousness), “礼” (li, ritual propriety), and “智” (zhì, wisdom), can different LLMs maintain semantic consistency?

Addressing this question is crucial for assessing the reliability of LLMs in the field of cultural transmission. Although traditional translation studies have profoundly revealed the variations in translations of such culture-loaded terms by different human translators, they have largely relied on qualitative comparisons, making it difficult to systematically capture patterns of variation at scale. Meanwhile, advancements in computational linguistics, particularly the maturation of sentence-level semantic similarity techniques based on sentence embeddings and automated tools for analyzing linguistic complexity (Norris and Ortega, 2009; Lu, 2010, 2011; McCarthy and Jarvis, 2010; Jarvis, 2013; Pallotti, 2015; Housen et al., 2019), offer new possibilities for quantifying semantic distances and structural features across different translations. While these methods have previously been employed to investigate the impact of machine translation on linguistic structural features (Lee, 2020; Chung and Ahn, 2022; Niu and Jiang, 2024), their application to assessing the semantic convergence of LLMs in translating cultural classics remains underexplored.

Currently, comparative studies on the translation of culturally dense texts, particularly Confucian classics, by multiple LLMs are still lacking. When different LLMs translate the same cultural concept, do they exhibit semantic “convergence,” reflecting commonalities in their training data, or do they demonstrate “divergence,” revealing differences in their internal semantic representations and strategic orientations? These questions remain systematically unanswered. To address this gap, this study takes *The Four Books* as a case study, integrating sentence embedding similarity analysis with linguistic complexity metrics to construct a multi-dimensional analytical framework for assessing translation stability. This framework aims to investigate the following three research questions:

RQ1: When translating highly culture-loaded texts such as *The Four Books*, do the English translations generated by different LLMs exhibit high overall cross-model semantic convergence?

RQ2: Against the backdrop of overall semantic stability, are there systematic differences in the semantic convergence of different core Confucian cultural concepts across translations by multiple models? Does this variation correlate with the semantic flexibility and interpretive space of these cultural concepts?

RQ3: When processing high-cultural-density contexts, do different LLMs display systematic patterns of semantic convergence or divergence? How do these patterns reflect differences in the models’ strategic orientations toward cultural semantic expression?

Beyond its methodological contribution to translation studies, this research engages with a broader question at the intersection of artificial intelligence and human cognition: Do LLMs, when processing culturally dense texts, converge upon a shared semantic space that mirrors or diverges from human conceptual organization? Recent work in computational neuroscience and AI has provided compelling evidence that language models trained on different languages converge onto similar embedding spaces, and that these model-derived spaces align with neural representations of meaning in the human brain (Xu et al., 2025). The “semantic hub hypothesis” further posits that models acquire a shared representational space across heterogeneous data types, placing semantically similar inputs near one another regardless of surface form (Wu et al., 2025). Within cognitive translatology, scholars have emphasized that human translators’ cognition, particularly their ability to access conceptual levels of meaning and generate multiple interpretive solutions, represents a key added value over disembodied artificial intelligence (Massey, 2021). These findings raise a critical, underexplored question for culturally loaded translation: If LLM translations exhibit cross-model semantic convergence, does this convergence reflect an emergent “algorithmic consensus” that aligns with human conceptual structures, or does it signal a homogenization of interpretive possibilities that diverges from the rich cognitive processing that culturally dense texts typically afford? By examining the semantic (in)stability of Confucian concept translation across multiple LLMs, this study provides an empirical foundation for addressing this question, bridging the gap between technical MT evaluation and cognitive translatology.

Regarding the research object, this study takes the classic cultural text of *The Four Books* as a holistic case. By focusing the analysis on its internal, highly loaded cultural semantic nodes (i.e., core concepts), it achieves both a micro-level exploration and a macro-level synthesis of semantic convergence in text translation. In terms of research methods, this study combines sentence embedding semantic similarity with various supplementary indicators to validate translation stability across multiple dimensions, providing a replicable analytical framework for empirical research in cultural translation. From a research perspective, through cross-model comparison, this study contributes to a deeper understanding of the characteristics of semantic representation when LLMs process cultural concepts. It should be noted that this study focuses on inter-model semantic convergence, and therefore the results should be interpreted as patterns of agreement rather than evidence of translation accuracy.

## 2 Literature review

Since the advent of neural machine translation in the artificial intelligence era in 2015, translation quality has seen a significant improvement (Li, 2021). Traditional NMT was initially based on the sequence-to-sequence (Seq2Seq) framework, with an encoder-decoder structure at its core, primarily accomplishing semantic mapping at the sentence-pair level (Sutskever et al., 2014; Shi et al., 2016). Subsequently, the integration of the attention mechanism effectively addressed the issue of long-distance dependencies, enabling dynamic alignment between the source and target languages and markedly enhancing the translation efficacy of long sentences (Bahdanau et al., 2015). The Transformer model, by employing a self-attention mechanism in place of recurrent neural network structures, significantly boosted parallel computing efficiency and became the mainstream architecture for NMT (Vaswani et al., 2017). In recent years, machine translation has continued to achieve breakthroughs in multilingual adaptation and model scaling (Wang et al., 2022). However, the performance of different NMT systems varies significantly in cross-genre translation (Li, 2021). The emergence of LLMs has propelled machine translation from sequence conversion toward generative language reconstruction (Jiao et al., 2023). Some large models can now scale to cover 200 languages, enabling massive multilingual translation (NLLB Team, 2024). Research indicates that ChatGPT outperforms some NMT systems in overall fluency and discourse coherence (AlAfnan, 2024; Son and Kim, 2023; Sizov et al., 2024).

However, issues of meaning drift and semantic generalization persist in the translation of cultural texts (Gao et al., 2024; Farghal and Haider, 2024). Wu (2023) also pointed out that when translating terms with Chinese characteristics, LLMs tend to adopt explanatory translation strategies, which may compromise the precision of cultural references in the source text. Furthermore, Vanmassenhove et al. (2019, 2021) introduced the concept of “machine translationese,” noting that algorithmic outputs exhibit structural simplification and linguistic convergence. While LLMs outperform NMT in term recognition, syntactic coherence, and semantic mapping in the translation of traditional Chinese medical classics, they struggle to adequately handle complex cultural semantics such as metaphors and philosophical connotations (Li et al., 2026). Evaluations focusing on culturally loaded texts like literary allusions further demonstrate that although large models possess advantages in knowledge accuracy and update efficiency, they still encounter issues such as knowledge mistranslation, information omission, and coarse language expression. This leads to crises of completeness, accuracy, transferability, and consistency at both the knowledge and linguistic levels (Han and Chai, 2024). They are also prone to problems like the loss of cultural connotations and the mechanical segmentation of semantics (Wang and Zhang, 2024; Wen and Tian, 2024).

Concomitant with the maturation of NMT technology, the academic community has gradually moved beyond assessing merely the surface-level quality of translations. It has become increasingly recognized that optimizing lexical alignment and model structures alone cannot guarantee semantic convergence and divergence. Traditional metrics like lexical diversity and syntactic complexity can only describe the formal linguistic features of translated texts, failing to directly measure semantic convergence and divergence. Moreover, increased linguistic complexity can exacerbate the model’s biases in deep semantic understanding and reproduction, with semantic ambiguity being particularly pronounced in the translation of culturally dense texts and abstract concepts. Against this backdrop, researchers have begun to focus on the issue of semantic convergence and divergence. Liu et al. (2024) introduced a bilingual-visual consistency constraint in multimodal NMT to strengthen translation coherence and consistency through cross-modal semantic alignment. Zhao and Wang (2025) pointed out that in multilingual translation, even if models achieve high accuracy at the lexical and syntactic levels, semantic drift and decreased consistency can still occur if they fail to effectively capture and reproduce deep semantic features, a problem especially evident in culturally loaded texts. Therefore, evaluating the translation quality of culturally dense texts necessitates moving beyond superficial linguistic indicators and constructing an evaluation framework centered on semantic preservation and transfer.

In the field of translation quality evaluation, research paradigms and technical paths have continuously evolved, yet related debates remain unresolved. Baker (1993, 1995) laid the groundwork for quantitative research using translation corpora, providing methodological support for comparative translation quality analysis. Traditional automatic evaluation metrics like BLEU and METEOR, while enabling efficient assessment based on n-gram matching, have inherent flaws in capturing semantic consistency (Chatzikoumi, 2020; Lee et al., 2023). With the advent of neural-based metrics such as COMET and BERTScore, research has begun to emphasize meaning similarity within semantic embedding spaces as a new evaluation dimension (Rivera-Trigueros, 2022). Concurrently, discussions on human-machine translation equivalence underscore the importance of evaluation design and the definition of measurement constructs (Läubli et al., 2020). Early research in translation quality assessment often focused on scoring consistency and reliability analysis (Stemler, 2019), emphasizing the measurement stability of evaluation results. With the advancement of computational linguistics, automatic evaluation methods have become mainstream, following a technological trajectory from n-gram matching to neural semantic matching (Han et al., 2021; Chauhan and Daniel, 2023). Wang et al. (2024) reviewed the strengths and weaknesses of three approaches, human evaluation, reference-based automatic evaluation, and reference-free automatic estimation, and proposed enhancing the precision of automatic evaluation, expanding its dimensions, and delving deeper into vertical domain assessment. Despite automatic evaluation becoming the dominant approach, existing metrics still struggle to fully capture the complexities of discourse coherence and cultural semantics (Han, 2020; Lee, 2020; Chung and Ahn, 2022; Rivera-Trigueros, 2022). For instance, the performance fluctuations of neural models are more pronounced under low-resource language conditions (Datta et al., 2023). Niu and Jiang (2024) noted that whether machine translation universally leads to simplification requires further validation across different genres and text types.

The translation of cultural classics involves abstract concepts, ethical terminology, and highly condensed semantic structures, possessing a linguistic density and philosophical depth far exceeding that of general discourse (Gao et al., 2024). In such texts, semantic convergence is reflected not only in lexical correspondence but also in the consistency of the conceptual system and the preservation of structural expressions. Although some studies have begun to examine the performance of LLMs in translating classical texts (Farghal and Haider, 2024), systematic cross-model comparative studies remain scarce, particularly those employing quantitative analysis based on semantic convergence. Consequently, there is a pressing need to delve deeper into the patterns of semantic convergence exhibited by LLMs when translating culturally dense texts, drawing from corpus-based and statistical perspectives. From the perspective of cultural semantics, the translation of culturally dense concepts involves more than lexical correspondence; it engages with what Levisen (2012) described as language-specific “universes of meaning.” Drawing on the Natural Semantic Metalanguage (NSM) framework, Levisen argued that cultural keywords function as privileged points of entry into the cognitive and social worlds of a linguistic community. This perspective resonates with the present study’s focus on Confucian core concepts, which likewise constitute a distinct semantic system whose cross-linguistic transfer cannot be reduced to simple denotational equivalence. Methodologically, Levisen’s emphasis on emic semantic analysis, capturing insider construals of meaning rather than expert-imposed definitions, parallels the present study’s decision to ground the analysis in the source text’s conceptual architecture rather than evaluating translations against a single authoritative rendering. Yet systematic investigation of how LLMs’ internal semantic representations of culturally specific concepts relate to human cognitive processing of these same concepts remains conspicuously absent. The present study addresses this gap by treating cross-model semantic convergence not merely as a metric of translation quality, but as a window into the emergent semantic organization of LLM systems, an organization that may carry implications for how human readers cognitively engage with machine-translated cultural classics.

## 3 Research methods

### 3.1 Experimental design

This study focuses on the semantic convergence characteristics exhibited by various LLMs when translating core concepts of Confucian culture from *The Four Books* into English. Unlike research aimed at translation quality assessment or model performance comparison, the primary concern of this study is not to judge translation quality, but rather to investigate whether different LLMs display convergent or divergent tendencies at the semantic level when processing identical cultural-linguistic input.

The research employs a mixed-methods design combining quantitative computation and qualitative analysis. Quantitatively, semantic vectors and cosine similarity calculations are used to construct a quantitative indicator of semantic convergence, depicting the degree of semantic proximity among translations from multiple models. Qualitatively, specific translation examples are examined to provide explanatory analysis of the quantitative results, revealing the underlying reasons for semantic divergence.

The specific procedure is as follows: First, the original Chinese text of *The Four Books* is segmented at the sentence level. Each original sentence is meticulously aligned with its corresponding English translations generated by different LLMs, creating a fully sentence-aligned parallel corpus covering the entire text. This approach maintains contextual integrity while ensuring computational manageability, achieving full-text coverage rather than localized sampling (Koehn, 2004). Second, based on a pre-trained semantic embedding model, all English translations are transformed into vector representations. For each source sentence, pairwise cosine similarities are computed among the English outputs generated by the four AI systems. The average of these six pairwise similarities serves as the sentence-level index of cross-model semantic convergence. This procedure yields a distribution of convergence scores across the entire text. Building on this, high-frequency, culturally loaded core concepts from *The Four Books* are extracted as analytical units. The sentence-level similarity results pertaining to sentences containing these concepts are aggregated to the conceptual level, characterizing the translational stability of different cultural semantic components. Finally, by integrating the full-text sentence-level distribution characteristics with the concept-level aggregated results, a comprehensive analysis is conducted on the representational differences among various models in conveying cultural semantics. The operational details of the design are distributed across the subsequent subsections for clarity: Section “3.2 Corpus sources” describes the source text, translation procedure, and parallel corpus construction. Section “3.3 Methods for analyzing semantic convergence” details the sentence embedding model, cosine similarity computation, and the extraction and disambiguation protocol for the twenty core Confucian concepts. The statistical comparison of pairwise model similarities, including effect size calculation and confidence interval estimation, is also specified in this section. Section “3.4 Validation against human reference translations” outlines the BERTScore validation against human reference translations.

### 3.2 Corpus sources

The research corpus selects the Confucian classics known as The Four Books (The Analects, Mencius, The Great Learning, and The Doctrine of the Mean). In terms of ideological content and linguistic expression, these texts centrally embody core concepts and value tenets of traditional Chinese culture, possessing high research representativeness. The experimental translations were generated by four mainstream LLMs: ChatGPT-5, Google Translate, Deepseek-V3.2, and Ernie Bot-5, covering diverse technological pathways and training backgrounds. To ensure the comparability of model outputs, a unified translation instruction was employed for all LLMs: “Please translate the following classical Chinese sentence into English.” The temperature parameter was set to 0 to minimize output randomness. Each sentence was translated once, without multiple sampling and averaging; Google Translate operated under its default translation mode. It is worth noting that Google Translate has historically been a pure NMT system, but since December 2025 it has integrated Gemini into its translation pipeline, transitioning toward a hybrid NMT-LLM architecture. The version accessed during data collection (early 2026) thus operates with LLM-powered contextual reasoning. Accordingly, we refer to the four systems collectively as “LLM-based translation systems,” while acknowledging their architectural heterogeneity.

A unified encoding framework can effectively integrate document-level contextual information, thereby enhancing cross-sentence semantic consistency (Ma et al., 2020). Therefore, all translated texts in this study were subjected to rigorous sentence-level alignment with the original text. This ensures that each analytical unit contains the same original Chinese sentence along with its corresponding translations from multiple models, providing a unified context for cross-model semantic comparison and safeguarding the validity of the experiment.

The Chinese source text of *The Four Books* was obtained from the Chinese Text Project^1^, a widely used open-access digital library of pre-modern Chinese texts. The specific editions used were: *The Analects* (Lunyu), *Mencius* (Mengzi), *The Great Learning* (Da Xue), and *The Doctrine of the Mean* (Zhong Yong), all based on the standard received versions with punctuation added by the digital editors. Prior to translation, the source text was segmented into independent sentences following the punctuation provided in the digital edition. Each sentence was treated as an isolated translation unit; no explicit chapter-level context was supplied to the LLMs beyond the sentence itself. While this decision may disadvantage models that benefit from broader discourse context, it ensures strict comparability across systems by holding the input constant and aligns with standard practice in sentence-level machine translation evaluation. The potential limitations of this decontextualized approach are addressed in Section “6 Conclusion.” For the alignment of human reference translations (see Section “4.4 Alignment with human translation traditions: BERTScore validation”), the public-domain English translations by James Legge (1861–1872) and the contemporary translation by Wu Guozhen (2012) were used.

### 3.3 Methods for analyzing semantic convergence

For semantic representation, evaluation methods based on semantic embeddings have been demonstrated to better reflect deep semantic consistency than traditional n-gram-based metrics (Rei et al., 2020). This paper employs a pre-trained sentence embedding model to vectorize the English translations, mapping each translated sentence into a unified semantic vector space. This numerically represents the semantic relationships among different translations, thereby providing the technical foundation for convergence analysis. In this study, semantic convergence specifically refers to “cross-model semantic consistency” the degree of convergence among English translations produced by different LLMs for the same source sentence within a high-level semantic representation space. This indicator solely measures the semantic proximity between different translations, without judging translation fidelity or fluency. As a preliminary verification step, source-to-target semantic similarity was also computed to confirm that all models generated outputs within a reasonable semantic range of the original Chinese. However, these scores are not the focus of this study and are not reported in the subsequent analysis, which concentrates exclusively on cross-model convergence in the target language.

The twenty core concepts analyzed in this study (Table 1a) were identified through a two-stage process. First, high-frequency content characters were extracted from the corpus. Second, the authors manually reviewed all occurrences of each candidate character to retain only those instances where the character functions as the relevant Confucian philosophical concept. For example, occurrences of 道 (dào) used as a verb meaning “to speak” were excluded; only nominal usages referring to the ethical or metaphysical “Way” were retained. Similarly, for 天 (tiân), occurrences within the political title 天子 (Son of Heaven) were excluded, while the compound 天下 (all under Heaven) was treated as a separate conceptual entry (see Table 1a). This manual filtering ensures that the subsequent stability analysis is based on semantically coherent instances of each Confucian term.

**TABLE 1a:** Distribution of the top 20 Confucian core concepts in *The Four Books*.

No.	Concept	Sentence count
1	王	284
2	道	259
3	民	246
4	仁	231
5	君子	229
6	天下	193
7	君	178
8	天	142
9	礼	134
10	义	117
11	心	116
12	德	100
13	政	96
14	中	95
15	贤	92
16	士	85
17	命	84
18	信	70
19	敬	66
20	治	58

**TABLE 1b:** Distribution of selected core concepts across *The Four Books*.

Concept	*The Great Learning*	*The Doctrine of the Mean*	*The Analects*	*Mencius*	Total
王	2	9	7	266	284
道	9	45	73	132	259
民	16	13	42	175	246
仁	8	6	88	129	231
君子	15	33	104	77	229
天下	6	22	21	144	193
君	2	2	41	133	178
天	3	34	23	82	142

Full distribution for all 20 concepts is available in the Supplementary Table 1.

**TABLE 2:** Semantic convergence of Confucian core concepts in multi-model translations of *The Four Books*.

No.	Concept	Mean cosine	Std	Sentence count
1	王	0.802	0.109	284
2	德	0.801	0.097	100
3	义	0.796	0.102	92
4	仁	0.795	0.096	231
5	敬	0.794	0.095	66
6	天	0.794	0.087	142
7	贤	0.791	0.101	91
8	政	0.790	0.117	95
9	天下	0.789	0.104	193
10	士	0.787	0.096	85
11	道	0.784	0.106	259
12	治	0.783	0.102	58
13	君	0.777	0.109	407
14	信	0.774	0.105	70
15	民	0.773	0.110	246
16	君子	0.770	0.100	229
17	中	0.766	0.111	94
18	命	0.752	0.127	84
19	礼	0.750	0.107	134
20	心	0.738	0.137	116

To ensure consistency and reproducibility in semantic representation, this study utilizes the all-mpnet-base-v2 sentence embedding model within the Sentence-BERT framework to encode the English translations. Sentences are mapped into a 768-dimensional semantic vector space, followed by mean pooling and L2 normalization to eliminate the interference of vector length differences on cosine similarity calculations. Cosine similarity is used to compute the semantic similarity between the translations of any two models [cosine(A,B) = (A⋅B)/(||A|| ||B||)]. The results from the six model-pair combinations are averaged to derive the sentence-level semantic convergence value. The embedding model employed is a general-purpose English semantic model and was not fine-tuned for specific tasks, thus avoiding adaptation bias toward particular model outputs.

To assess whether the observed differences in pairwise semantic similarity among model pairs (Table 3) were statistically significant, we conducted paired-samples *t*-tests on the sentence-level cosine similarity scores. Specifically, for each of the six model pairs, the vector of sentence-level similarity scores was compared against that of the reference pair (DeepSeek–Ernie Bot). Given the large number of sentences, the *t*-test is robust to moderate violations of normality. However, we acknowledge that sentences within the corpus are not strictly independent observational units, as they are nested within chapters and thematic contexts. To mitigate this concern and to provide a more nuanced picture of the practical magnitude of the observed differences, we supplement the *p*-values with Cohen’s d effect sizes and 95% confidence intervals for the mean differences. Cohen’s d is interpreted according to conventional thresholds: | d| < 0.2 (negligible), 0.2 ≤ | d| < 0.5 (small), 0.5 ≤ | d| < 0.8 (medium), and | d| ≥ 0.8 (large). All statistical analyses were performed in Python using the scipy.stats and pingouin libraries.

**TABLE 3:** Pairwise similarity results between models.

Model pair	Mean cosine similarity	Std	Mean diff.	Cohen’s d	95% CI of diff.
ChatGPT-5-Google Translate	0.761	0.184	−0.058	−0.33	[−0.063, −0.052]
ChatGPT-5-Deepseek-V3.2	0.776	0.155	−0.024	−0.17	[−0.028, −0.020]
ChatGPT-5-Ernie Bot-5	0.764	0.159	−0.033	−0.24	[−0.037, −0.029]
Google Translate-Deepseek-V3.2	0.706	0.183	−0.096	−0.60	[−0.101, −0.091]
Google Translate-Ernie Bot-5	0.710	0.185	−0.092	−0.55	[−0.097, −0.087]
Deepseek-V3.2-Ernie Bot-5	0.805	0.144	–	–	–

At the data aggregation level, all sentences containing the same core cultural concept are grouped into an analytical set. The semantic similarity results are summarized using means and standard deviations, forming a concept-level semantic convergence indicator and enabling an integrated analysis from individual translation cases to the conceptual level. The validity of this indicator is underpinned by distributed semantics theory and a construct validity framework: cosine similarity of sentence vectors can effectively characterize the degree of semantic convergence in texts, possessing theoretical plausibility. Concurrently, pairwise semantic similarities between models are calculated to compare their convergent or divergent characteristics in processing cultural semantics.

To avoid misinterpreting “semantic convergence” as “cultural fidelity,” this study incorporates qualitative translation example analysis to elucidate phenomena such as semantic generalization or the weakening of cultural connotations. It is emphasized that semantic convergence here represents a structural characterization within the distributed semantic space, rather than a value judgment on translation quality.

### 3.4 Validation against human reference translations

To address the question of whether cross-model semantic convergence correlates with alignment to established human translational norms and to provide a meaningful benchmark for interpreting the cosine similarity thresholds reported in Section “4 Results,” we conducted an auxiliary evaluation using BERTScore. BERTScore computes token-level semantic similarity between a candidate translation and a reference translation using contextual embeddings from pre-trained language models. Unlike n-gram-based metrics such as BLEU, BERTScore has been shown to correlate strongly with human judgments of semantic adequacy, particularly for literary and culturally dense texts.

For each of the four constituent texts of *The Four Books* (*The Great Learning*, *The Doctrine of the Mean*, *The Analects*, and *Mencius*), we calculated the BERTScore F1 between every LLM-generated translation and two authoritative human reference translations:

(1) James Legge (1861–1872): A 19th-century missionary translation widely regarded as foundational in Anglophone Confucian studies. Legge’s renderings are characterized by a formal, often theistic interpretive framework and are extensively represented in public-domain digital corpora.
(2) Wu Guozhen (2012): A contemporary scholarly translation that reflects modern Sinological conventions and strives for terminological consistency while remaining accessible to general readers. Wu’s translation is under copyright and is less likely to have been included in LLM training data.

BERTScore was computed using the microsoft/deberta-xlarge-mnli model, which has demonstrated robust performance across diverse semantic evaluation tasks. The F1 scores reported herein represent the harmonic mean of precision and recall, with values theoretically ranging from 0 to 1, where higher scores indicate greater semantic overlap with the reference.

Rationale for BERTScore over cosine similarity with references: while the primary analysis (Section “4.2 Semantic convergence in the translation of culture-loaded words”) employs cosine similarity of sentence embeddings to measure inter-LLM convergence, a direct cosine comparison between LLM outputs and human references would be confounded by differences in sentence segmentation, syntactic style, and lexical choice. BERTScore, by operating at the token level with soft semantic matching, provides a more robust and interpretable measure of alignment with human translational norms.

The results of this validation serve two purposes in the present study: (a) they establish whether the LLM translations fall within the semantic range of accepted human renderings; and (b) they provide an external benchmark against which the inter-LLM convergence scores (Table 2) can be contextualized. Specifically, the human-human BERTScore (Legge vs. Wu) establishes a natural baseline of translational variance that helps interpret whether an inter-LLM similarity should be considered “high” or “low” in the context of *The Four Books*.

## 4 Results

### 4.1 Distributional characteristics of culture-loaded words

This study employs the culture-loaded words of *The Four Books* as the entry point for semantic analysis, a choice grounded in sound scholarly rationale. The philosophical system of *The Four Books* is highly condensed, with core concepts such as “仁” (rén), “义” (yì), “礼” (li), and “智” (zhì) possessing dual attributes as both lexical units and semantic hubs. They carry multiple layers of meaning encompassing Confucian ethics, cognition, and socio-politics, serving as crucial semantic anchors within this culturally dense text. This study does not treat these concepts as isolated objects of inquiry but rather as a structured reference for examining overall translation stability. Their level of abstraction, semantic openness, and distributional features directly determine the robustness and explanatory scope of the stability analysis.

As shown in Table 1a, the selected core cultural concepts exhibit significant variation in their frequency of occurrence within the corpus, which aligns with the heterogeneous thematic structure of *The Four Books*. Crucially, each concept has a sufficient sample size to effectively avoid statistical biases associated with small samples, thereby ensuring the robustness and comparability of cross-model semantic convergence calculations. Furthermore, the variance in concept frequency provides a foundation for interpreting subsequent results: high-frequency concepts can reveal the overall tendency of models when processing recurring cultural information, while concepts with lower frequencies, though representative, better illustrate the translation strategies models employ for specific cultural semantics. These data characteristics lay the necessary groundwork for the analysis of semantic convergence.

The distribution of these concepts across the four constituent texts of *The Four Books* is provided in Table 1b for a representative subset. Following the same counting protocol as Table 1a, the values represent the number of sentences in which each concept appears, not raw character frequency. The four texts share a common Confucian thematic core, yet differ substantially in length and genre: *The Great Learning* (110 sentences) and *The Doctrine of the Mean* (243 sentences) are short, aphoristic treatises, whereas *The Analects* (1,293 sentences) and *Mencius* (2,330 sentences) are longer dialogic works. Table 1b reveals marked unevenness in concept distribution.

### 4.2 Semantic convergence in the translation of culture-loaded words

Having established the coverage of each core concept within the corpus, this paper takes the semantic units anchored by these cultural concepts as the analytical starting point to investigate the semantic convergence of translations produced by different models. Semantic convergence is defined here as the degree of similarity in the semantic vector space among translations of sentences corresponding to the same cultural concept from different models. It serves to quantify the cross-model semantic convergence and divergence in translations.

As shown in Table 2, the cross-model average similarity for the 20 core Confucian concepts is consistently above 0.73, indicating an overall semantic consensus among LLMs in translating this classic text. However, the stability of different concepts exhibits significant stratification (ranging from 0.734 to 0.802), which is negatively correlated with the concept’s level of abstraction and semantic complexity. The high-stability group (mean > 0.79), represented by “王” (wáng), “德” (dé), “义” (yì), and “仁” (rén), features clearly defined semantic boundaries and established cross-linguistic equivalents. The medium-stability group (0.76–0.79), including concepts like “道” (dào), “君” (jûn), “民” (mín), and “君子” (jûnzi), combines concrete reference with abstract connotations, leading to diverging translation strategies. The low-stability group (mean < 0.76), comprising “命” (mìng), “礼” (li), and “心” (xîn), represents the most central and abstract philosophical concepts in Confucianism. “命” (mìng) involves heavenly mandate and fate; “礼” (li) is a complex of rituals, norms, and inner feelings; “心” (xîn) integrates cognitive, emotional, and moral functions. These concepts have almost no precise equivalents in English, possessing high semantic flexibility and interpretative space, which results in lower translation similarity and higher dispersion.

**FIGURE 1:**
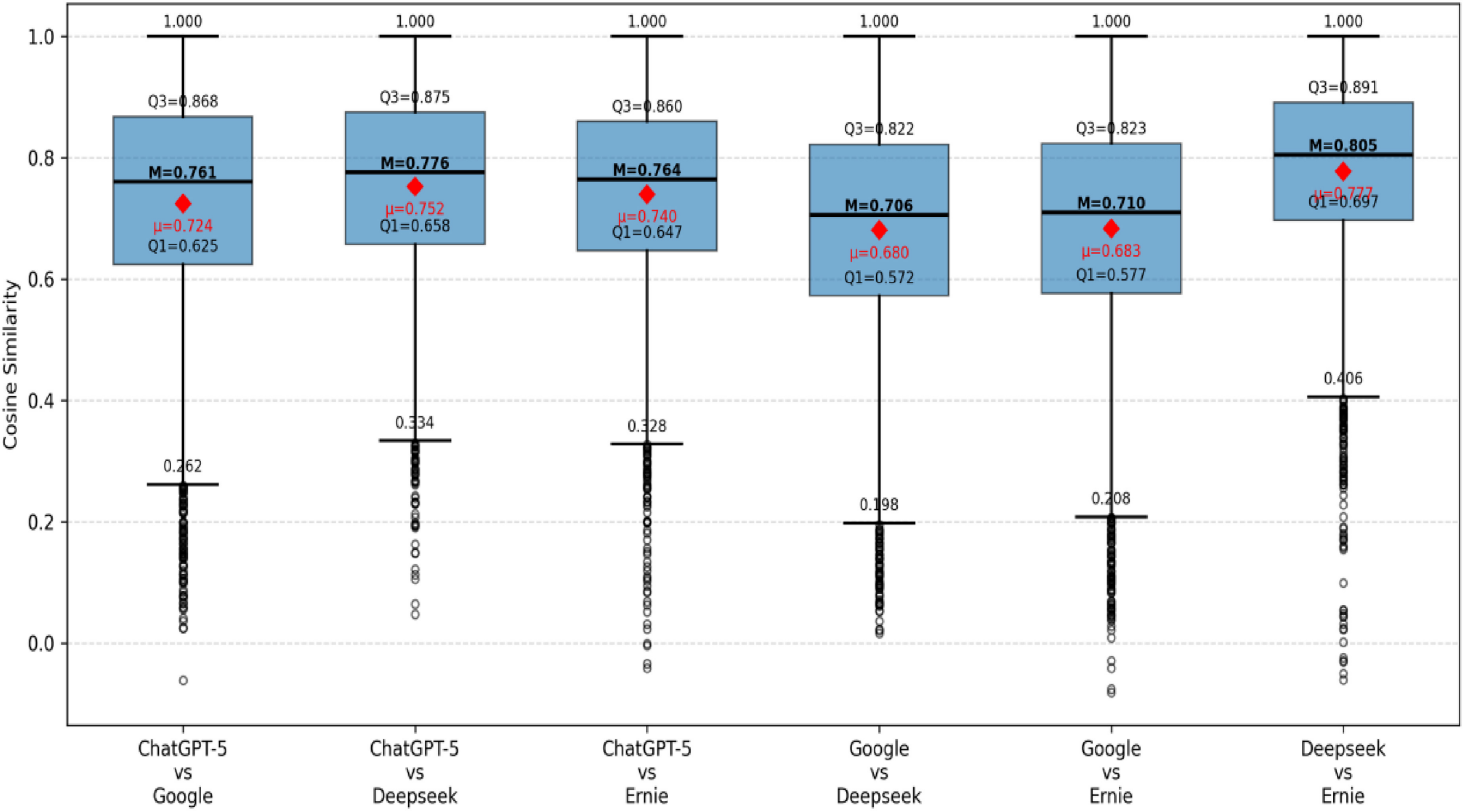
Pairwise semantic similarity between translation models.

Figure 1 shows that all model pairs demonstrate relatively high semantic convergence, with medians ranging from 0.706 to 0.805 and all upper quartiles exceeding 0.82. This indicates a high degree of overlap in the semantic space among multi-model translations within this culturally dense context, making cross-model semantic convergence the primary trend. Notably, the Deepseek–Ernie pair exhibits the highest overall consistency (median = 0.805; Q1 = 0.687), suggesting a robust foundational semantic alignment. The two pairs involving Google Translate show relatively lower medians (0.706 and 0.710) and lower first quartiles (Q1 = 0.57), indicating weaker consistency in lower-similarity contexts. The three pairs involving ChatGPT-5 fall within an intermediate stability range (medians between 0.761 and 0.776), with relatively balanced interquartile structures, demonstrating overall semantic convergence while allowing for moderate contextual flexibility. Importantly, all distributions exhibit a pronounced negative skew, with extended lower tails and a number of low-similarity outliers. This suggests that despite the strong overall semantic convergence, model-specific semantic divergence still occurs concentratedly under certain contextual conditions. These deviations are not merely statistical noise but represent structural manifestations of models adopting different interpretative pathways when navigating highly dense cultural contexts.

Overall, the semantic convergence of translated culture-loaded words exhibits a stratified structure of macroscopic convergence and microscopic divergence, resulting from the interaction between the abstract properties of cultural concepts and the generative mechanisms of the models.

### 4.3 Comparison of semantic similarity among different LLMs

Building on the concept-level stability differences, this study calculates pairwise semantic similarity between models and employs statistical significance testing to examine the semantic proximity of different models in translating cultural concepts. Figure 1 and Table 3 complement each other: Figure 1 presents the overall distribution of semantic convergence for model pairs, while Table 3 quantifies the relative differences in semantic expression between models.

Table 3 presents pairwise semantic similarity statistics among the four LLM systems, including mean cosine similarity, standard deviations, and– relative to the most convergent pair (DeepSeek–Ernie)– mean differences, Cohen’s d effect sizes, and 95% confidence intervals. The DeepSeek–Ernie pair exhibits the highest mean similarity (*M* = 0.805, Std = 0.144), serving as the reference group for subsequent comparisons. All other model pairs show lower mean similarities, with differences reaching statistical significance (all 95% CIs exclude zero). However, the practical magnitude of these differences varies substantially. The pairs involving ChatGPT yield small effect sizes (| d| = 0.17–0.33), indicating that while their divergence from the reference pair is detectable given the large sample size, it is substantively modest. In contrast, the pairs involving Google Translate yield medium effect sizes (*d* = −0.55 and −0.60), suggesting a more meaningful degree of semantic divergence from the most convergent pair. The non-overlapping confidence intervals between the ChatGPT-involving and Google-involving pairs further support the inference that Google Translate occupies a more distinct semantic region in its renderings of *The Four Books*. These effect size estimates provide a more nuanced interpretation than *p*-values alone. The significant differences for all pairs are largely driven by the large sample size; the Cohen’s d values reveal that only the Google Translate pairs exhibit divergence of a magnitude that may be considered practically meaningful. This pattern aligns with the distributional characteristics observed in Figure 1, where Google Translate pairs display notably lower medians and greater dispersion.

### 4.4 Alignment with human translation traditions: BERTScore validation

To contextualize the cross-model convergence scores reported in Section “4.2 Semantic convergence in the translation of culture-loaded words” and to provide an external benchmark for interpreting their magnitude, we conducted a BERTScore evaluation against two authoritative human translations: James Legge and Wu Guozhen. BERTScore computes token-level semantic similarity between a candidate translation and a reference, yielding an F1 score that correlates strongly with human judgments of semantic adequacy.

The detailed BERTScore results are presented in Table 4. The BERTScore results confirm that all LLM translations fall within a range conventionally associated with high semantic adequacy (F1 > 0.75). DeepSeek consistently achieves the highest alignment with the human references, while Ernie Bot yields the lowest mean score. Notably, the ranking of models by BERTScore (DeepSeek > Google ≈ ChatGPT > Ernie Bot) shows broad correspondence with their pairwise cross-model convergence patterns (see Section “4.2 Semantic convergence in the translation of culture-loaded words”), wherein DeepSeek and Ernie Bot formed the most convergent pair and Google Translate exhibited greater divergence from other models. It is important to note that BERTScore F1 and the cosine similarity metric employed in Section “4.2 Semantic convergence in the translation of culture-loaded words” operate on different semantic scales and are not directly numerically comparable. The BERTScore validation is intended solely to establish that the LLM outputs are semantically grounded in the human translation tradition, not to provide a direct quantitative benchmark for the inter-LLM similarities.

**TABLE 4:** BERTScore F1 between LLM translations and human references.

Model	*The Great Learning*	*The Doctrine of the Mean*	*The Analects*	*Mencius*	Mean
DeepSeek	0.913	0.925	0.859	0.886	0.896
Google	0.845	0.85	0.821	0.884	0.852
ChatGPT	0.844	0.824	0.823	0.845	0.834
Ernie Bot	0.784	0.826	0.859	0.844	0.829

## 5 Discussion

### 5.1 Translation convergence of highly stable cultural concepts

Analysis of the data in Table 2 reveals gradient differences in the translation stability of various cultural-semantic components within *The Four Books*. Core concepts like “王” (wáng) and “德” (dé) demonstrate average cosine similarity values significantly higher than the overall mean, indicating robust semantic convergence. The reason for this lies in the clear referential objects and relatively fixed cross-cultural correspondences of such concepts, leading different translation models toward noticeable convergence in their semantic output.

**Table:** 

Example 1	ST:	子曰：“无忧者，其唯文王乎？”
	TT:	ChatGPT-5: Confucius said: “Was not King Wen free from anxiety?” Google Translate: Confucius said: “The one who was without worry was King Wen, wasn’t he?” Deepseek-V3.2: The Master said, “It is only King Wen of whom it can be said that he was without anxiety.” Ernie Bot-5: The Master said, “Is there anyone free from worry? It must have been King Wen.”

In this example, all four models uniformly translate “文王” as the historical figure “King Wen.” Although there are minor syntactic and phrasing differences, they maintain a high degree of consistency regarding the referent, semantic focus, and evaluative tone. This highly convergent translation directly explains the concept’s prominent performance in the stability metrics.

**Table:** 

Example 2	ST:	庸德之行，庸言之谨，有所不足，不敢不勉，有余不敢尽。
	TT:	ChatGPT-5: Practice ordinary virtue, speak ordinary words with caution; if there is something lacking, one dares not fail to make an effort; if there is something extra, one dares not exhaust it. Google Translate: In matters of ordinary conduct and cautious speech, if there is something insufficient, one dares not fail to strive; if there is something in excess, one dares not use it up. Deepseek-V3.2: Earnest in practicing ordinary virtues, and careful in ordinary speech; if there is any deficiency, he dares not neglect to exert himself; if there is any surplus, he dares not fully expend it. Ernie Bot-5: In practicing common virtue and being careful in ordinary speech, if there is any shortcoming, one dares not fail to make effort; if there is any excess, one dares not exhaust it.

Here, “德” (dé) is almost consistently rendered as “virtue/virtues” across models, anchoring it as a practicable ethical quality. Although the Google Translate output shows some lexical omission, the overall semantics still revolve around “moral practice and self-restraint,” without substantial divergence. While such convergent translations yield high cross-model similarity scores, this convergence should not be conflated with cultural fidelity. The uniform rendering of “德” as “virtue” across all models may reflect a shared bias in training data toward standardized, decontextualized equivalents, rather than a nuanced grasp of the concept’s ethical and ritual dimensions in classical Confucian discourse. High agreement does not guarantee accuracy; it may instead signal a collective flattening of semantic complexity.

### 5.2 Translation divergence of low-stability cultural concepts

In stark contrast to “王” (wáng) and “德” (dé), cultural concepts such as “心” (xîn), “礼” (li), and “道” (dào) exhibit low semantic convergence. Such concepts are typically characterized by a high degree of abstraction or multiple layers of cultural meaning, making it easier for different models to activate divergent interpretative pathways during comprehension and expression, leading to semantic divergence.

**Table:** 

Example 3	ST:	欲修其身者，先正其心。
	TT:	ChatGPT-5: Those who wish to cultivate themselves must first rectify their mind. Google Translate: Those who want to cultivate themselves must first correct their hearts. Deepseek-V3.2: He who wishes to cultivate himself must first set right his mind. Ernie Bot-5: Those who desire to cultivate their character must first rectify their heart.

In this example, “心” (xîn) is rendered by different models as either “mind” or “heart.” The former leans toward a psychological or cognitive understanding, while the latter carries more pronounced emotional or ethical overtones. This difference directly impacts the overall semantic focus of the sentence and explains the lower similarity score for this concept in the stability calculations.

**Table:** 

Example 4	ST:	武王末受命，周公成文武之德，追王大王、王李，上祀先公以天子之礼。
	TT:	ChatGPT-5: King Wu had not yet fully received the mandate when the Duke of Zhou completed the virtue of Kings Wen and Wu, posthumously honored King Tai and King Ji, and offered sacrifices to former lords with the rites of the Son of Heaven. Google Translate: When King Wu did not receive the mandate, the Duke of Zhou completed the virtues of King Wen and King Wu, and posthumously honored King Da and King Li, and offered sacrifices to the former dukes with the rites due to the Son of Heaven. Deepseek-V3.2: It was in his old age that King Wu received the appointment to the throne, and the Duke of Zhou completed the virtuous course of Wen and Wu. He carried up the title of king to Tai and Ji, and sacrificed to all the former dukes with the ceremonies of the Son of Heaven. Ernie Bot-5: King Wu received the Mandate late in life, but the Duke of Zhou perfected the virtue of Kings Wen and Wu, posthumously honored Tai Wang and Wang Ji as kings, and sacrificed to the former dukes with the rites of the Son of Heaven.”

In this case, “礼” (li) is translated variously as “rites,” “rituals,” and “ceremonies.” While these terms belong to the same semantic field, they differ in their emphasis on institutionality, religiosity, or ceremonial formality. Such subtle but consistent divergences further widen the semantic distance between model outputs.

**Table:** 

Example 5	ST:	天命之谓性，率性之谓道，修道之谓教。
	TT:	ChatGPT-5: What Heaven has endowed is called human nature; to follow that nature is called the Way; to cultivate the Way is called instruction. Google Translate: What Heaven ordains is called human nature; to follow one’s nature is called the Way; to cultivate the Way is called teaching. Deepseek-V3.2: What Heaven has conferred is called Nature; following Nature is called the Path; cultivating the Path is called instruction. Ernie Bot-5: What is ordained by Heaven is called nature; following nature is called the Way; cultivating the Way is called education.

In this example, while translations for “道” (dào) such as “Way” and “Path” belong to the same semantic field, they exhibit noticeable interpretative flexibility. These divergent phenomena confirm that the higher the abstraction and semantic complexity of a cultural concept, the lower the cross-linguistic translational consistency and, consequently, the weaker its semantic convergence.

### 5.3 Divergent semantic strategies of different translation models in culturally loaded texts

The boxplot (Figure 1) and pairwise model similarity results (Table 3) indicate that the semantic convergence of translations for culturally loaded texts is influenced not only by the characteristics of the cultural concepts themselves but also by the overall semantic generation strategies of the models. Some model pairs show a concentrated distribution of semantic similarity with higher medians and narrower box ranges, suggesting a tendency toward abstract, interpretative translation strategies. This approach achieves cross-model convergence by downplaying cultural imagery and reinforcing universal semantics. Conversely, other model pairs exhibit longer lower whiskers and greater dispersion, stemming from strategic differences between “cultural preservation” and “functional explanation”: some models adhere closely to the source language’s cultural imagery and conceptual structure, while others prioritize target language comprehensibility through syntactic restructuring and semantic explication.

The high similarity between DeepSeek and ERNIE Bot may be attributable to overlapping training corpora or similar alignment objectives. One plausible hypothesis is that both models, developed by Chinese technology companies, were exposed to large volumes of stylistically consistent Chinese-English parallel texts–such as canonical translations of Chinese classics–leading them to converge on similar lexical and syntactic choices. However, whether this convergence constitutes cultural preservation or merely reflects a shared data-induced bias cannot be determined from similarity scores alone.

Google Translate’s lower similarity to other models may reflect its optimization toward broad cross-linguistic fluency rather than adherence to source-culture textual conventions. This tentative interpretation aligns with its observed tendency toward syntactic simplification and lexical generalization in the present dataset. Further investigation, including qualitative analysis of translation strategies and examination of model training objectives, would be required to substantiate this claim.

It is crucial to note that these inter-model semantic differences are not direct indicators of translation quality but rather systematic phenomena resulting from the interaction between the semantic flexibility of cultural concepts and model generation mechanisms, not random noise. Through comparative analysis at the model level, we can better understand the overall orientations of different machine translation systems in handling cultural semantics, providing necessary context for subsequent in-depth discussion of outliers and specific translation examples.

### 5.4 Typological analysis of boxplot outliers: sources of cultural semantic noise

In Figure 1, numerous outliers significantly deviating from the main distribution are observable. These outliers are predominantly concentrated at the lower end of the similarity distribution, manifested as extended lower whiskers and a high number of discrete points. Through manual verification and classification analysis, the primary sources of these outliers can be categorized into four types: semantic divergence from polysemous words, differential handling of personal names and institutional cultural references, translational shifts due to strategic differences, and overall semantic shifts caused by contextual misjudgment.

The first type of outlier mainly originates from the numerous polysemous words prevalent in the Chinese Confucian classics. These words can carry abstract philosophical meanings in some contexts while referring to specific persons, social roles, or behavioral norms in others, with their semantic boundaries being inherently highly open.

**Table:** 

Example 6	ST:	抑而强与？
	TT:	ChatGPT-5: Or your own kind of strength?” Google Translate: Suppressing and forcing?

In this sentence, “抑” (yì) can function as a conjunction meaning “or,” or as a verb meaning “to suppress” or “to restrain.” “强” (qiáng/qiang) can be a noun meaning “strength” or a verb meaning “to force.” ChatGPT-5 interprets “抑” as “or,” rendering the entire sentence as a choice relationship. Google Translate interprets both words verbally, rendering an action relationship (“suppressing and forcing”). Both interpretations are grammatically plausible, but their semantic directions are entirely different.

**Table:** 

Example 7	ST:	思戢用光。
	TT:	ChatGPT-5: Bows and arrows were stretched, weapons were readied on the battlefield. Google Translate: Think of using up light. Deepseek-V3.2: He sought to gather and glorify his people.

“戢” (jí) has two meanings: “to store away weapons” and “to gather/assemble.” “光” (guâng) can mean “glory” or “light.” ChatGPT-5 adopts “store weapons” and “light,” extending to the gleam of weapons on a battlefield. Google Translate renders it literally as “use up light.” Deepseek-V3.2 chooses “gather” and “glory.” The three translations thus point toward three distinct semantic fields: military, physical, and governance.

The second type of outlier involves differences in handling personal names, place names, and specific cultural references. *The Four Books* contain numerous pre-Qin figures, state names, and institutional titles for which no single, universally accepted English equivalent exists. Among the outlier sentences, some models tend toward transliteration or pinyin transcription, while others use free translation or interpretive paraphrasing.

**Table:** 

Example 8	ST:	嬖奚反命曰：“天下之良工也。”
	TT:	Google Translate: The favored concubine retorted, “He is the finest craftsman in the world.” Deepseek-V3.2: Xi reported back, saying, “He is the most incompetent driver in the world.”

“嬖奚” (Bì Xî) is a personal name (Xi, a favored minor official). Google Translate misinterprets “嬖” as “favored concubine” and renders “良工” as “finest craftsman.” Deepseek-V3.2 correctly identifies “奚” as a name via transliteration but mistranslates “良工” as “most incompetent driver” (the opposite meaning). This involves both differences in handling proper nouns (mistranslation vs. transliteration) and a contextual misunderstanding of “良工” (a skilled craftsman/officer). Notably, such outliers are not concentrated in any single model but are distributed across different model pairs, suggesting their origin lies more in the inherent cross-linguistic asymmetry of cultural proper nouns than in simple model capability gaps.

**Table:** 

Example 9	ST:	彻者，彻也；助者，藉也。
	TT:	ChatGPT-5: To clear is clearing; to assist is aiding. Ernie Bot-5: “Che” means “to share”; “zhu” means “to assist.”

This sentence is Mencius explaining an ancient tax system. “彻” (chè) and “助” (zhù) are technical terms. ChatGPT-5 uses free translation, employing general verbs. Ernie Bot-5 uses a “transliteration + paraphrase” strategy, preserving the terminological feel. The two approaches lead to divergence in terminological consistency.

The third type of outlier reflects differences at the level of translation strategy, particularly the divergence between interpretive translation and relatively literal translation. Some original sentences are highly condensed and structurally simple, but a purely literal English translation often fails to convey their implicit ethical or philosophical nuances. In such cases, some models tend to preserve the original structure, minimally converting words, while others add explanatory components to make the implicit logic explicit. This strategic divergence does not necessarily constitute a conflict in core semantic understanding, but due to differences in sentence length, information density, and explicit semantic structure, their vector representations can show significantly lower similarity. Looking at the outlier distribution, this type appears across various model pairs, indicating it reflects divergences in translation decision-making rather than model-specific random errors.

**Table:** 

Example 10	ST:	中立而不倚，强哉矫！
	TT:	ChatGPT-5: He stands upright without leaning–how strong is this! Google Translate: To remain neutral and impartial is truly admirable!

Both convey the core idea of being upright and unbiased. However, ChatGPT-5 adopts a literal translation plus exclamation (“stands upright without leaning”), preserving the original imagery. Google Translate abstracts it to “remain neutral and impartial” and uses “truly admirable” for the exclamation “强哉矫.” This strategic difference results in differing expressive styles and semantic emphasis.

**Table:** 

Example 11	ST:	有弗学，学之弗能，弗措也；有弗问，问之弗知，弗措也；有弗思，思之弗得，弗措也；有弗辨，辨之弗明，弗措也；有弗行，行之弗笃，弗措也。
	TT:	Deepseek-V3.2: The superior man, while there is anything he has not studied, or while in what he has studied there is anything he cannot understand, will not intermit his labor. While there is anything he has not inquired about, or anything in what he has inquired about which he does not know, he will not intermit his labor. While there is anything which he has not reflected on, or anything in what he has reflected on which he does not apprehend, he will not intermit his labor. While there is anything which he has not discriminated, or his discrimination is not clear, he will not intermit his labor. If there be anything which he has not practiced, or his practice fails in earnestness, he will not intermit his labor. Ernie Bot-5: If there is something not studied, study it until you can; if there is something not asked, ask until you know; if there is something not thought of, think until you get it; if there is something not discriminated, discriminate until it is clear; if there is something not practiced, practice it until it is firm.

Both models accurately grasp the original text’s parallel structure and core meaning (“unless studied, do not stop until proficient”). The difference lies purely in syntactic strategy. Deepseek constructs complex sentences conforming to formal English style, retaining the abstract subject “superior man” and the verb “intermit.” Ernie Bot-5 opts for explicating the logic, breaking it into conditional-result clauses using the more concrete “until” structure for greater clarity. This difference between abstraction and concreteness is the primary driver of the semantic vector dissimilarity.

The fourth type of outlier can be attributed to local contextual misjudgment or translational drift. In complex contexts like literary texts, LLMs may produce critical semantic misreadings or coherence errors. This type typically manifests when a model fails to accurately identify a sentence’s function within the larger discourse or misinterprets archaic Chinese structures such as ellipsis or inversion.

**Table:** 

Example 12	ST:	不识有诸?
	TT:	ChatGPT-5: Did this happen? Google Translate: Do you not know of their existence?

This sentence is from *Mencius: King Hui of Liang I*, where Mencius asks King Xuan of Qi if he has heard of a certain matter (like the story of killing an ox with a knife). ChatGPT-5 translates it as “Did this happen?” pointing to whether the event occurred. Google Translate renders it as “Do you not know of their existence?” pointing to knowledge of the event’s existence. Neither accurately captures the nuance of “having heard of something,” but they misjudge the context in different directions.

**Table:** 

Example 13	ST:	自取之也。
	TT:	ChatGPT-5: This is what one brings upon himself. Google Translate: He brought it upon himself. Deepseek-V3.2: It is all determined by the water itself.

This sentence is from *Mencius: Li Lou II*, discussing that water’s tendency to flow downward is due to its natural propensity. ChatGPT-5 and Google Translate both understand it as “self-inflicted,” losing the connection to the “water” context. Deepseek-V3.2 correctly associates it with “the water itself,” but the expression remains somewhat vague. Overall, the models fail to grasp that “取之” here metaphorically refers to water’s tendency to seek lower ground.

However, proportionally, such outliers constitute a relatively limited fraction of all low-similarity sentences and are not concentrated in any single model. This suggests that while LLMs still have limitations in comprehending Classical Chinese, their grasp of the overall semantic structure remains relatively reliable.

In summary, the outliers presented in Figure 1 are not statistically accidental noise but result from the interplay of multiple linguistic and cultural factors. Their presence does not undermine the overall semantic convergence revealed earlier; instead, it corroborates the “coexistence of stability and divergence” characteristic of cross-model translation of classical texts. They reflect the different pathways and expressive tendencies different models employ in understanding Confucian cultural concepts, providing crucial support for deeper investigation into the stability and variability of cultural concept translation.

### 5.5 Interpreting convergence in light of human reference alignment

The BERTScore validation (Section “4.4 Alignment with human translation traditions: BERTScore validation”) establishes that the LLM translations are not semantically aberrant; they consistently achieve moderate to high alignment with two authoritative human translations. This finding mitigates the concern that the cross-model convergence documented in Section “4.2 Semantic convergence in the translation of culture-loaded words” might reflect random or incoherent outputs. However, the relationship between inter-LLM convergence and human-reference alignment is not straightforward.

First, the ranking of models by BERTScore roughly parallels their positioning in the cross-model similarity analysis. DeepSeek, which exhibited the highest pairwise similarities with other models (Figure 1), also achieves the highest BERTScore against human references. Conversely, Ernie Bot, despite its strong convergence with DeepSeek, shows the lowest BERTScore, suggesting that its semantic space, while proximate to DeepSeek’s, is comparatively more distant from the human translational mainstream.

Second, the fact that all models achieve BERTScore F1 > 0.78, values that in prior work have been associated with translations of good semantic adequacy, indicates that the LLM outputs are viable English renderings of *The Four Books*. Yet the persistent cross-model divergences documented in Section “5.2 Translation divergence of low-stability cultural concepts” (e.g., “心” as “mind” vs. “heart”) demonstrate that this broad adequacy coexists with unresolved interpretive pluralism. In other words, LLMs can produce translations that are simultaneously acceptable and divergent.

The findings of this study can be interpreted through the lens of the “semantic hub hypothesis” (Wu et al., 2025), which posits that language models learn a shared representation space across heterogeneous data types, placing semantically similar inputs near one another regardless of surface form. The high cross-model convergence observed for concrete referential terms like 王 (“king”) and 德 (“virtue”) suggests that LLMs have internalized stable, averaged representations of these concepts, representations that may align with the centers of meaning that human translators and readers also access. From a psycholinguistic perspective, such convergence could theoretically facilitate comprehension by presenting consistent conceptual mappings across different machine-generated renderings. However, the opposite interpretation is equally plausible: the algorithmic consensus may actually impoverish the interpretive richness that human readers expect from culturally dense texts, potentially leading to shallower cognitive engagement. This concern resonates with recent findings that structured, human-like conceptual representations can emerge purely from language prediction in LLMs, but that such representations may lack the sensorimotor grounding that characterizes fully embodied human concepts (Xu et al., 2025).

The marked divergence observed for abstract concepts like 心 (heart-mind), 礼 (ritual propriety), and 道 (the Way), where models oscillate between “mind” and “heart,” “rites” and “ceremonies” resonates with cognitive translatology’s emphasis on human translators’ ability to generate multiple target-text solutions from a single conceptual level (Massey, 2021). That different LLMs make systematically different choices here (see Section “5.2 Translation divergence of low-stability cultural concepts”) suggests that these models, despite lacking embodied experience, may nonetheless be sensitive to the interpretive problem space that such abstract concepts present. The divergence is not mere noise; it is a structural manifestation of unresolved semantic ambiguity at the algorithmic level, ambiguity that mirrors, in a rudimentary form, the interpretive deliberations that characterize expert human translation. From a cognitive standpoint, this divergence may signal to human readers that a given term carries multiple legitimate interpretations, potentially prompting deeper cognitive processing, a phenomenon akin to “desirable difficulty” in the learning sciences.

The present findings underscore the need for a new generation of translation evaluation metrics that move beyond surface-level similarity to assess interpretive adequacy, that is, whether a translation affords the same range of cognitive and interpretive possibilities as the source text. Recent work on cognitively informed multi-agent translation frameworks (Li et al., 2025) demonstrates that incorporating cognitive processes such as drafting, refinement, and context reasoning can enhance translation quality, suggesting that LLM translation may benefit from more explicit modeling of human-like cognitive strategies. Future research should incorporate human perception studies that directly measure reading fluency, comprehension accuracy, and cognitive load when engaging with convergent versus divergent LLM translations of Confucian concepts. Such work would help adjudicate whether algorithmic convergence facilitates or inadvertently constrains the rich cognitive processing that culturally dense texts are uniquely positioned to afford.

Thus, the BERTScore validation contextualizes rather than directly benchmarks the cross-model convergence scores. The inter-LLM similarities (0.73–0.80) should not be interpreted as either “high” or “low” in relation to the BERTScore values; they are simply different measures capturing distinct facets of semantic relationship. What the combined evidence underscores is that algorithmic convergence in the LLM ecosystem is a relative, not absolute, phenomenon, one that operates within a broader semantic landscape defined by the human translation tradition but does not collapse into it.

## 6 Conclusion

This study investigated the English translations of the Confucian classic *The Four Books*, focusing on core culturally loaded concepts. Utilizing a sentence-aligned corpus and combining quantitative measurement via sentence vector cosine similarity with qualitative analysis, it systematically explored the characteristics of semantic convergence in cross-model translations by LLMs. The findings reveal that: English translations of *The Four Books* produced by different LLMs exhibit a pronounced tendency toward cross-model semantic convergence, with outputs clustering tightly in vector space around shared representational patterns. Notably, this convergence does not necessarily imply greater fidelity to the source text’s cultural semantics; rather, it points to an emergent algorithmic consensus whose relationship to human translational traditions warrants further scrutiny; the semantic convergence of cultural concepts is not uniformly distributed but exhibits gradient differences based on their level of abstraction and interpretative space, with the inherent semantic flexibility of a concept being a key factor influencing translational stability; and different models exhibit systematic strategic divergences in cultural translation, with inter-model differences stemming from strategic choices in conveying cultural meaning rather than failures in core semantic comprehension. Cases of low semantic similarity predominantly reflect inherent challenges in cultural translation, such as handling polysemous words, converting cultural proper nouns, and differing strategic orientations.

This study reveals the underlying principles governing LLMs’ processing of Chinese classic translations from the perspective of semantic convergence, providing empirical evidence for understanding the emergent properties of cross-linguistic transfer in culturally loaded texts processed by AI systems. At the same time, the observed divergence in low-stability concepts and the unverified relationship between convergence and accuracy underscore the need for caution when deploying LLMs for the translation and dissemination of culturally dense classics. Future research should triangulate these computational findings with expert human evaluation to determine whether algorithmic convergence facilitates or inadvertently erodes the transmission of nuanced cultural meaning. This research focused solely on core concepts within *The Four Books* and primarily examined the semantic dimension, presenting certain limitations, which include the exclusive focus on core concepts and semantic dimensions, the decontextualized sentence-level input ill-suited to discourse-dependent Classical Chinese, and the absence of direct human cognitive validation, leaving the link between algorithmic convergence and psycholinguistic processing inferential.

Future research could expand the scope to include more Chinese classics and cultural traditions, integrate multi-dimensional evaluations encompassing style, rhetoric, and discourse ideology, and combine human expert assessment with multi-feature quantitative analysis to more comprehensively deconstruct the generative logic of LLMs in cultural translation. Crucially, future work should also incorporate psycholinguistic measures, such as reading comprehension tests, cognitive load assessments, and eye-tracking studies. To empirically validate whether the convergence and divergence patterns documented here have measurable effects on how human readers cognitively engage with machine-translated Confucian classics. Such work would deepen the interdisciplinary study of intelligent translation and the global dissemination of Chinese culture from both computational and cognitive perspectives.

## Data Availability

The original contributions presented in this study are included in this article/Supplementary material, further inquiries can be directed to the corresponding author.

## Footnotes

1
https://ctext.org
