# Supplementary material for: Semantic convergence in culturally loaded text translation by Large Language Models: a cross-model empirical analysis of English translations of The Four Books
Source: Front Psychol. 2026 May 14;17:1829488. doi: 10.3389/fpsyg.2026.1829488 (PMC13215814; doi:10.3389/fpsyg.2026.1829488)
Supplement: Supplementary file 1 [file Supplementary_file_1.docx]

**Supplementary Materials**

TABLE S1. Distribution of the 20 core Confucian concepts across the four books of *The Four Books*

| **Concept** | **The Great Learning** | **The Doctrine of the Mean** | **The Analects** | **Mencius** | **Total Sentence Count** |
| --- | --- | --- | --- | --- | --- |
| 王 | 2 | 9 | 7 | 266 | 284 |
| 道 | 9 | 45 | 73 | 132 | 259 |
| 民 | 16 | 13 | 42 | 175 | 246 |
| 仁 | 8 | 6 | 88 | 129 | 231 |
| 君子 | 15 | 33 | 104 | 77 | 229 |
| 天下 | 6 | 22 | 21 | 144 | 193 |
| 君 | 2 | 2 | 41 | 133 | 178 |
| 天 | 3 | 34 | 23 | 82 | 142 |
| 礼 | 0 | 16 | 57 | 61 | 134 |
| 义 | 3 | 2 | 23 | 89 | 117 |
| 心 | 9 | 0 | 6 | 101 | 116 |
| 德 | 10 | 20 | 34 | 36 | 100 |
| 政 | 0 | 6 | 40 | 50 | 96 |
| 中 | 3 | 17 | 21 | 54 | 95 |
| 贤 | 2 | 7 | 22 | 61 | 92 |
| 士 | 0 | 4 | 16 | 65 | 85 |
| 命 | 6 | 6 | 23 | 49 | 84 |
| 信 | 2 | 7 | 34 | 27 | 70 |
| 敬 | 3 | 6 | 22 | 35 | 66 |
| 治 | 7 | 6 | 4 | 41 | 58 |
